# Clinical courses and predictors of left ventricular systolic dysfunction in systemic sclerosis: A cohort study

**DOI:** 10.1515/rir-2024-0014

**Published:** 2024-07-15

**Authors:** Jakrapan Werakiat, Burabha Pussadhamma, Ajanee Mahakkanukrauh, Siraphop Suwannaroj, Chingching Foocharoen

**Affiliations:** Department of Medicine, Faculty of Medicine, Khon Kaen University, Khon Kaen, Thailand

**Keywords:** systemic sclerosis, scleroderma, systolic dysfunction, myocarditis, cohort study

## Abstract

**Background and Objectives:**

Left ventricular systolic dysfunction (LVSD) is a cardiac involvement that is the leading cause of death among patients with systemic sclerosis (SSc). We aimed to define the clinical course and predictors of LVSD among SSc patients.

**Methods:**

We conducted a cohort study among adult patients with SSc who were followed up from 2013 to 2020. Semiparametric Cox regression analysis with robust clustering by cohort identification number was used to evaluate the predictors of LVSD.

**Results:**

Among the 3, 987 person-years, LVSD was defined in 35 of 419 SSc patients for an incidence of 0.88 per 100 person-years. The median duration of the disease was 8.5 (interquartile range (IQR) 4.9–12.9) years. Every 1-point increase in the modified Rodnan skin score (mRSS) and salt and pepper skin were strong predictors of LVSD, with a respective adjusted hazard ratio (HR) of 1.05 and 3.17. During follow-up, 26 cases (74.3%) had unimproved LVSD. The strong predictors of the unimprovement of LVSD were every 1-point increase in mRSS (HR 1.05), every 1 mg increase in prednisolone treatment (HR 1.05), and every 1 U/L increase in creatine kinase (CK) (HR 1.001). Mycophenolate treatment was a protective factor against the unimprovement of LVSD in SSc (HR 0.15).

**Conclusions:**

LVSD was frequently found in patients with diffuse cutaneous SSc, and in most cases, it remained unimproved during follow-up. High mRSS, steroid use, and high CK levels were predictors of unimproved LVSD, whereas mycophenolate treatment might prevent the progression of LVSD. Steroids should be prescribed with caution in patients with longer disease duration.

## Introduction

Systemic sclerosis (SSc) is a rare connective tissue disease. SSc is classified into 2 major groups: limited cutaneous systemic sclerosis (lcSSc) and diffused cutaneous systemic sclerosis (dcSSc).^[1]^ The majority of Thai patients suffer from dcSSc.^[2]^

Involvement of the heart in SSc can lead to serious outcomes. Cardiac involvement is reportedly associated with high mortality in patients with SSc.^[3]^ Any part of the heart can be involved. Pericardial involvement is characterized by fibrinous pericarditis and chronic effusion.^[4,5]^ Cardiac tamponade is rare.^[4]^ Mitral valve prolapse is reported to be the most frequent form of endocardial involvement.^[6,7]^ Myocardial involvement includes microvascular ischemia, patchy fibrosis unrelated to coronary distribution, myocarditis, septum, and posterior wall, or asymmetrical septal hypertrophy without systemic arterial hypertension. Myocarditis is also detected by cardiac magnetic resonance imaging (MRI) in SSc patients independent of inflammatory or cardiac markers.^[8]^ Due to manifestation heterogeneity, the prevalence of primary cardiac involvement is difficult to determine.^[7]^

Primary cardiac involvement in SSc is frequent, although the early disease may be asymptomatic.^[9]^ Left ventricular (LV) diastolic dysfunction is a common cardiac involvement in SSc and is often asymptomatic.^[9]^ Long-term LV diastolic dysfunction is reported to be associated with dyspnea and a decline in functional status in the Australian Scleroderma Cohort, although no significant association with heart failure and mortality has been found.^[10]^ While LV systolic dysfunction, another cardiac involvement in SSc, is associated with high mortality and diminished physical capability in patients with SSc.^[11]^ According to the high mortality related to cardiac involvement, particularly LV systolic dysfunction among SSc patients, no predictor of LV systolic dysfunction in SSc has been defined. If predictors of LV systolic dysfunction are identified, interventions acting on these modifiable predictors might improve patient outcomes. Therefore, we sought to identify the clinical course and predictors of LV systolic dysfunction in SSc, as evaluated by conventional echocardiography.

## Methods

This cohort study included adult patients with SSc who attended and were followed up at the Scleroderma Clinic, Srinagarind Hospital, Khon Kaen University, Khon Kaen, Thailand, between January 2013 and December 2020. We included patients over 18 years of age who (a) fulfilled the 2013 Classification Criteria for Systemic Sclerosis: American College of Rheumatology/European League Against Rheumatism Collaborative Initiative^[12]^ and (b) had undergone echocardiography at least 2 times during follow-up either for screening or for diagnosis. We excluded patients with (a) overlap syndrome;(b) previous history of congestive heart failure, pulmonary hypertension, or defined as LV systolic dysfunction before a diagnosis of SSc; (c) potential causes of LV systolic dysfunction, such as viral infection, coronary artery disease, alcohol-related, stress-induced, familial cardiomyopathy, thiamine deficiency, thyrotoxicosis, and peripartum; (d) structural heart disease that can reduce ejection fraction, such as aortic or mitral valve disease; and, (e) a history of taking medicines or substances that can cause LV systolic dysfunction, such as doxorubicin and cocaine.

### Operational Definitions

SSc subsets are classified into lcSSc and dcSSc by the LeRoy classification.^[1]^ LV systolic dysfunction is defined as LV ejection fraction (LVEF) measured by conventional echocardiography less than 50% with or without cardiac symptoms.^[13,14]^ The clinical course of LV systolic dysfunction with or without treatment at the last follow-up was classified into two groups. The first is the improvement of LV systolic dysfunction, defined as improvement of LVEF with a return of > 50%. Second, unimproved LV systolic dysfunction characterized by declining LVEF compared to the first detection of LV systolic dysfunction, where the LVEF was < 50% during follow-up, or the patient died.

The start date was when the patient had any initial non-Raynaud SSc symptoms. Pulmonary fibrosis was considered present when either chest radiography or high-resolution computed tomography (HRCT) detected interstitial fibrosis. Pulmonary arterial hypertension (PAH) was diagnosed when the mean pulmonary arterial pressure (mPAP) was > 20 mmHg at rest with a pulmonary artery wedge pressure of ≤ 15 mmHg and a pulmonary vascular resistance of ≥ 3 Wood units, as confirmed by right heart catheterization.^[15]^ Esophageal involvement was defined when any esophageal symptoms of SSc were present (*i.e*., esophageal dysphagia, heartburn, or reflux symptoms). Stomach involvement was characterized by early satiety or vomiting.^[16]^ Intestinal involvement was determined if any of the following were present: diarrhea, bloating, malabsorption, constipation, ileus, or pseudo-intestinal obstruction. Muscle weakness was defined as either proximal muscle weakness or generalized weakness as detected by physicians. Steroid administration was divided into three groups according to the prednisolone equivalency:(a) high dose was > 30 mg/day; (b) moderate dose > 7.5 but ≤ 30 mg/day; and, (c) low dose ≤ 7.5 mg/ day.^[17]^

### Statistical Analysis

The clinical characteristics were categorized as continuous or categorical. Continuous data are presented as means and standard deviations or as medians and interquartile ranges (IQR), as appropriate. Categorical data are presented as proportions or percentages. Semi-parametric Cox regression analysis (with robustness cluster by the cohort identification number of the patient) was used to assess the predictors of LV systolic dysfunction and unimprovement of LV systolic dysfunction. The hazard ratio and 95% confidence interval between the predictors, LV systolic dysfunction, and unimprovement of LV systolic dysfunction were assessed. Hazard ratios that accounted for the effects of several variables (*i.e*., variables with *P*-value < 0.1) were entered into a Cox regression model. Variables were tested for significance using the Wald χ^2^ test, and all statistical tests were two-tailed. Statistical significance was set at *P* < 0.05. All data analyses were performed using STATA version 16.0 (StataCorp., Texas, USA).

## Results

**Figure 1:**
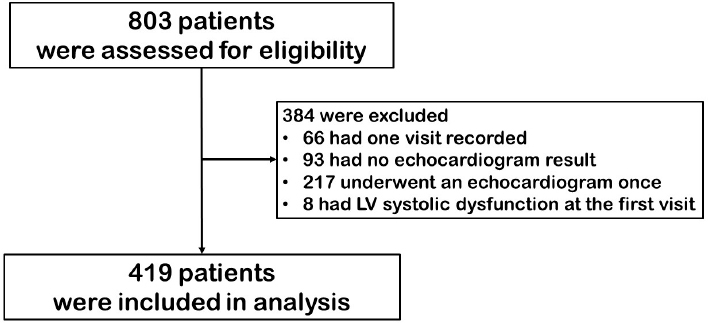
Flow of patients. LV, left ventricular.

A total of 10, 268 visits of 803 SSc patients from the SSc database registry were assessed for potential eligibility. Three hundred and eighty-four patients were excluded according to the exclusion criteria: only one visit (66 cases), no echocardiography data (93 cases), no follow-up echocardiographic data (217 cases), and LV systolic dysfunction at the first visit (8 cases). The flow chart of the patients is presented in Figure 1.

The remaining 419 patients with SSc were included in this analysis. The mean age of onset was 49.6±11.5 years; 66.1% were female, and 69.9% had the dcSSc subset. The median duration of the disease was 8.5 (IQR 4.9–12.9) years. Demographic data and clinical characteristics of the 419 patients with SSc at the last visit are presented in Table 1.

**Table 1:** Demographic data.

Data	*N* = 419
Female (%)	277 (66.1)
Age of onset (years), mean ± SD	49.6 ± 11.5
Age at last follow-up (years), mean ± SD	59.1 ± 10.5
Duration of follow-up (years), median (IQR)	8.5 (4.9-12.9)
dcSSc subset (%)	292 of 418 (69.9)
WHO functional class;(%)	
I	128 of 306 (41.8)
II	133 of 306 (43.5)
III	38 of 306 (12.4)
IV	7 of 306 (2.3)
mRSS (point); median (IQR)	2 (0-8)
Raynaud’s phenomenon (%)	186 (44.4)
Digital ulcer (%)	76 (18.1)
Digital gangrene (%)	10 (2.4)
Telangiectasia (%)	178 (42.5)
Calcinosis cutis (%)	36 (8.6)
Salt and pepper skin (%)	165 (39.4)
Edematous skin (%)	24 (5.7)
Tendon friction rub (%)	55 (13.1)
Hand deformity (%)	167 (39.9)
Arthritis (%)	26 (6.2)
Muscle weakness;(%)	12 (2.9)
Esophageal involvement (%)	168 (40.1)
Stomach involvement (%)	63 (15.0)
Intestinal involvement (%)	74 (17.7)
Pulmonary fibrosis (%)	206 of 418 (49.3)
Pulmonary arterial hypertension (%)	82 of 416 (19.7)
Hemoglobin (g/dL), mean ± SD	12.1 ± 4.3
Creatinine (mg/dL), median (IQR)	0.8 (0.6-1.0)
Albumin (g/dL), mean ± SD	4.1 ± 0.8
CRP (mg/L), median (IQR)	4.3 (2.2-29.0)
Creatinine kinase (U/L), median (IQR)	99 (64-158)

WHO, World Health Organization; SD, standard deviation; IQR, Interquartile range; dcSSc, diffuse cutaneous systemic sclerosis; ESR, erythrocyte sedimentation rate; CRP, C-reactive protein.

During the follow-up period of 3, 987 person-years, 35 patients were defined as having LV systolic dysfunction with an incidence rate of 0.88 per 100 person-years (95%CI: 0.63–1.22), while 35 patients had LV systolic dysfunction, 23 (65.7%) females, and 26 cases (74.3%) had the dcSSc subset (Supplemental Table 1).

According to the univariate analysis, factors associated with LV systolic dysfunction in patients with SSc were (a) high modified Rodnan skin score, (b) salt and pepper skin, (c) tendon friction rub, and (d) muscle weakness. The factors associated with LV systolic dysfunction in the univariate analysis are presented in Table 2.

**Table 2:** Predictors associated with LV systolic dysfunction.

Data	HR (95% CI)	*P*-value
Female	0.91 (0.43-1.94)	0.81
Age (years)	0.99 (0.94-1.04)	0.73
dcSSc subsets	2.14 (0.97-4.73)	0.06
mRSS (points)	1.07 (1.04-1.10)	<0.001^*^
Raynaud’s phenomenon	1.71 (0.97-2.97)	0.06
Digital ulcer	1.15 (0.56-2.38)	0.70
Digital gangrene	0.94 (0.24-3.73)	0.92
Telangiectasia	0.92 (0.50-1.69)	0.79
Calcinosis	0.47 (0.11-2.04)	0.32
Salt and pepper skin	3.68 (2.03-6.69)	<0.001^*^
Edematous skin	1.59 (0.58-4.36)	0.37
Tendon friction rub	2.77 (1.29-5.94)	0.009^*^
Hand deformity	1.14 (0.60-2.18)	0.69
Arthritis	0.64 (0.15-2.71)	0.55
Muscle weakness	4.26 (1.83-9.88)	<0.001^*^
Esophageal involvement	1.25 (0.68-2.30)	0.47
Stomach involvement	0.99 (0.42-2.31)	0.98
Intestinal involvement	0.58 (0.28-1.12)	0.14
Pulmonary fibrosis	0.83 (0.46-1.47)	0.52
Pulmonary hypertension	1.37 (0.68-2.77)	0.38
Hemoglobin (g/dL)	1.02 (0.99-1.04)	0.054
Creatinine (mg/dL)	1.00 (0.94-1.05)	0.87
Albumin (g/dL)	0.54 (0.24-1.25)	0.15
CRP (mg/L)	1.00 (0.99-1.00)	0.78
Creatinine kinase (U/L)	1.00 (1.00-1.00)	0.05

^*^Statistical significant. dcSSc, diffuse cutaneous systemic sclerosis; mRSS, modified Rodnan skin score; WHO, World Health Organization; ESR, erythrocyte sedimentation rate; CRP, C-reactive protein.

According to the semiparametric Cox regression analysis with cluster robustness, there were two models for predicting LV systolic dysfunction in patients with SSc. Model 1 included creatine kinase levels, while Model 2 included muscle weakness. Both models showed that every 1-point increase of mRSS and salt and pepper skin were strong predictors of LV systolic dysfunction with respective adjusted hazard ratio (HR) of 1.05 (95%CI: 1.01–1.09) and 3.17 (95%CI: 1.40–7.22) in model 1 and 1.04 (95%CI: 1.01–1.07) and 2.37 (95%CI: 1.20–4.69 in model 2. At the same time, muscle weakness was an additional predictor of LV systolic dysfunction in model 2 with adjusted HR 2.74 (95%CI: 1.21–6.20). The predictors of LV systolic dysfunction evaluated using a semiparametric Cox regression analysis with cluster robustness by the identification number (ID) of the patients are presented in Table 3.

**Table 3:** Predictors of LV systolic dysfunction analysis by semiparametric cox regression analysis with robustness cluster by the ID of the patients.

Data	Crude HR (95%CI)	Adjusted HR (95%CI)
Model 1		
dcSSc subset	2.14 (0.97-4.73)	2.12 (0.79-5.65)
Every 1-point increase in mRSS	1.07 (1.04-1.10)	1.05 (1.01-1.08)^*^
Raynaud’s phenomenon	1.71 (0.97-2.97)	1.29 (0.70-2.39)
Salt and pepper skin	3.68 (2.03-6.69)	3.17 (1.59-6.32)^*^
Tendon friction rub	2.77 (1.29-5.94)	1.03 (0.45-2.35)
Every 1-U/L increase in creatine kinase	1.00 (1.00-1.00)	1.0002 (0.99-1.001)
Model 2		
dcSSc subset	2.14 (0.97-4.73)	1.25 (0.60-2.59)
Every 1-point increase in mRSS	1.07 (1.04-1.10)	1.04 (1.01-1.07)^*^
Raynaud’s phenomenon	1.71 (0.97-2.97)	1.31 (0.76-2.26)
Salt and pepper skin	3.68 (2.03-6.69)	2.37 (1.31-4.30)^*^
Tendon friction rub	2.77 (1.29-5.94)	1.48 (0.73-3.03)
Muscle weakness	4.26 (1.83-9.88)	2.74 (1.15-6.52)^*^

^*^Statistical significant. dcSSc, diffuse cutaneous systemic sclerosis; mRSS, modified Rodnan skin score; LV, left ventricular.

Among those with LV systolic dysfunction, 26 (74.3%) patients had unimproved LV systolic dysfunction or died. Sixteen of the 26 cases (61.5%) were female, and 21 (80.8%) had the dcSSc subset. The demographic data of the last visit of the patients with LV systolic dysfunction are presented in Table 4.

**Table 4:** Demographic data of the last visit of patients with LV systolic dysfunction.

Data	Improvement of LV systolic dysfunction *N* = 9	Unimprovement of LV systolic dysfunction *N* = 26	*P*-value
Female (%)	7 (77.9)	16 (61.5)	0.45
Age of onset (years), mean ± SD	51.5 ± 10.9	51.5 ± 10.1	0.99
Age on the date of LV systolic dysfunction detection (years), mean ± SD	58.5 ± 9.8	57.6 ± 9.0	0.78
Age at last follow-up (years), mean ± SD	62.2 ± 9.8	58.8 ± 8.6	0.32
Duration of disease on the date of LV systolic dysfunction detection (years), mean ± SD	7.0 ± 5.4	6.1 ± 4.3	0.56
Duration of follow-up (years), median (IQR)	10.7 ± 5.4	7.3 ± 5.0	0.10
Time interval between baseline and the last follow-up echocardiography (years), mean ± SD	9.7 ± 1.9	6.5 ± 0.9	0.11
dcSSc subset (%)	5 (55.6)	21 (80.8)	0.19
WHO functional class (%)			0.04^*^
I	5 of 8 (62.5)	3 of 20 (15.0)	
II	2 of 8 (25.0)	13 of 20 (65.0)	
III	1 of 8 (12.5)	4 of 20 (20.0)	
IV	0	0	
mRSS (points), median (IQR)	2 (0-5)	5 (2-21)	0.07
Raynaud’s phenomenon (%)	2 (22.2)	19 (73.1)	0.02^*^
Digital ulcer (%)	0	7 (26.9)	0.15
Digital gangrene (%)	0	2 (7.7)	0.99
Telangiectasia (%)	4 (44.4)	14 (53.9)	0.71
Calcinosis cutis (%)	0	3 (11.5)	0.55
Salt and pepper skin (%)	4 (44.4)	17 (65.4)	0.43
Edematous skin (%)	0	3 (11.5)	0.55
Tendon friction rub (%)	0	11 (42.3)	0.03^*^
Hand deformity (%)	3 (33.3)	12 (46.2)	0.70
Arthritis (%)	0	2 (7.7)	0.99
Muscle weakness (%)	0	2 (7.7)	0.99
Esophageal involvement (%)	4 (44.4)	15 (57.7)	0.70
Stomach involvement (%)	1 (11.1)	6 (23.1)	0.65
Intestinal involvement (%)	3 (33.3)	4 (15.4)	0.34
Pulmonary fibrosis (%)	5 (55.6)	10 (38.5)	0.45
Pulmonary arterial hypertension (%)	3 (33.3)	9 (34.6)	0.99
Hemoglobin (g/dL), mean ± SD	11.8 ± 1.2	11.6 ± 2.2	0.82
Creatinine (mg/dL), median (IQR)	1 (0.9-1.2)	0.9 (0.7-1.0)	0.12
Albumin (g/dL), mean ± SD	3.9 ± 0.7	3.7 ± 0.6	0.34
CRP (mg/L), median (IQR)	0.8 (0.8-0.8)	27.1 (1.2-53)	0.22
Creatinine kinase (U/L), median (IQR)	74 (69-103)	164 (52-120)	0.21
Treatment			
Aspirin (%)	6 of 6 (100)	17 of 17 (100)	0.99
Nifedipine (%)	6 (66.7)	13 (50.0)	0.65
Domperidone (%)	5 (55.6)	13 (50.0)	0.99
Cyclophosphamide (%)	0	5 of 26 (19.2)	0.30
Mycophenolate mofetil (%)	3 (33.3)	2 (7.7)	0.10
Hydroxychloroquine (%)	1 (11.1)	1 (3.8)	0.45
Prednisolone (%)	5 (55.6)	19 (73.1)	0.49
Low dose	3	7	
Moderate dose	2	12	
High dose	0	0	

^*^Statistical significant. SD, standard deviation; IQR, Interquartile range; dcSSc, diffuse cutaneous systemic sclerosis; WHO, World Health Organization; ESR, erythrocyte sedimentation rate; CRP, C-reactive protein; LV, left ventricular.

According to the univariate analysis, factors associated with the unimprovement of LV systolic dysfunction in SSc patients were (a) male sex, (b) high modified Rodnan skin score, (c) salt and pepper skin, (d) edematous skin, (e) tendon friction rub, (f) muscle weakness, (g) high creatine kinase, and (h) receiving prednisolone at any dose. The factors associated with unimprovement of LV systolic dysfunction using univariate analysis are presented in Table 5.

**Table 5:** Predictors associated with the unimprovement of LV systolic dysfunction.

Data	HR (95% CI)	*P*-value
Female	0.41 (0.17-0.95)	0.04^*^
Age (years)	0.99 (0.95-1.04)	0.88
dcSSc subsets	0.99 (0.39-2.55)	0.99
mRSS (points)	1.07 (1.04-1.10)	<0.001^*^
Raynaud’s phenomenon	1.35 (0.85-2.15)	0.20
Digital ulcer	1.06 (0.58-1.93)	0.84
Digital gangrene	0.80 (0.42-1.51)	0.49
Telangiectasia	0.77 (0.46-1.27)	0.31
Calcinosis	0.98 (0.44-2.16)	0.95
Salt and pepper skin	1.77 (1.02-3.09)	0.04^*^
Edematous skin	3.27 (1.62-6.60)	0.001^*^
Tendon friction rub	1.81 (1.09-3.02)	0.02^*^
Hand deformity	0.98 (0.48-2.00)	0.95
Arthritis	0.85 (0.32-2.26)	0.75
Muscle weakness	2.31 (1.31-4.08)	<0.004^*^
Esophageal involvement	1.30 (0.70-2.44)	0.40
Stomach involvement	0.81 (0.42-1.54)	0.53
Intestinal involvement	0.91 (0.48-1.74)	0.78
Pulmonary fibrosis	0.88 (0.49-1.60)	0.68
Pulmonary hypertension	0.63 (0.34-1.14)	0.13
Hemoglobin (g/dL)	1.00 (0.94-1.05)	0.92
Creatinine (mg/dL)	0.73 (0.36-1.47)	0.38
Albumin (g/dL)	0.85 (0.52-1.37)	0.52
CRP (mg/L)	1.00 (0.99-1.00)	0.23
Creatinine kinase (U/L)	1.002 (1.001-1.002)	<0.001^*^
Treatment		
Aspirin (%)	0.98 (0.74)	0.91
Nifedipine (%)	0.93 (0.43-2.05)	0.86
Domperidone (%)	1.04 (0.64-1.68)	0.86
Cyclophosphamide (%)	2.09 (1.01-4.33)	0.05
Mycophenolate mofetil (%)	2.06 (0.04-1.09)	0.06
Hydroxychloroquine (%)	2.49 (0.41-15.29)	0.32
Prednisolone (%)	2.15 (1.23-3.75)	0.01^*^
Prednisolone dose; every 1 mg increasing	1.08 (1.04-1.12)	<0.001^*^
Low dose	1.93 (1.09-3.41)	0.02^*^
Moderate dose	2.40 (1.26-4.59)	0.01^*^
High dose	-	-

^*^Statistical significant. dcSSc, diffuse cutaneous systemic sclerosis; mRSS, modified Rodnan skin score; WHO, World Health Organization; ESR, erythrocyte sedimentation rate; CRP, C-reactive protein.

According to the semiparametric Cox regression analysis with cluster robustness, there were two models for predicting the unimprovement of LV systolic dysfunction in SSc. Model 1 included creatine kinase, while model 2 included muscle weakness. In both models 1 and 2, each 1-point increase in mRSS and every 1-mg addition of prednisolone were strong predictors of unimproved LV systolic dysfunction, with adjusted HRs of 1.05 (95%CI: 1.01–1.07) and 1.05 (95%CI: 1.02–1.09) in model 1, and 1.05 (95%CI: 1.02–1.08) and 1.06 (95%CI: 1.02–1.10) in model 2. In addition, every 1-U/L increase in creatine kinase was also a predictor of unimprovement LV systolic dysfunction in Model 1 (Table 5). By contrast, mycophenolate mofetil was a protective factor against the unimprovement of LV systolic dysfunction in model 2 with adjusted HR 0.15 (95%CI: 0.02–0.93). The predictors of unimprovement of LV systolic dysfunction evaluated by the semiparametric Cox regression analysis with cluster robustness by the ID of the patients are presented in Table 6.

**Table 6:** Predictors associated with unimproved LV systolic dysfunction analysis by semiparametric cox regression analysis with robustness cluster by the ID of the patients.

Data	Crude HR (95%CI)	Adjusted HR (95%CI)
Model 1		
Female	0.41 (0.17-0.95)	0.67 (0.30-1.48)
mRSS	1.07 (1.04-1.10)	1.05 (1.02-1.07)^*^
Salt and pepper skin	1.77 (1.02-3.09)	1.14 (0.75-1.74)
Edematous skin	3.27 (1.62-6.60)	1.29 (0.69-2.42)
Tendon friction rub	1.81 (1.09-3.02)	0.98 (0.63-1.50)
Every 1-U/L increase in creatine kinase	1.002 (1.001-1.002)	1.001 (1.0002-1.002)^*^
Cyclophosphamide	2.09 (1.01-4.33)	0.96 (0.44-2.09)
Mycophenolate mofetil	2.06 (0.04-1.09)	0.19 (0.03-1.33)
Every 1-mg increase of prednisolone	1.08 (1.04-1.12)	1.05 (1.02-1.09)^*^
Model 2		
Female	0.41 (0.17-0.95)	0.59 (0.25-1.39)
mRSS	1.07 (1.04-1.10)	1.05 (1.02-1.08)^*^
Salt and pepper skin	1.77 (1.02-3.09)	1.07 (0.70-1.63)
Edematous skin	3.27 (1.62-6.60)	1.34 (0.68-2.64)
Tendon friction rub	1.81 (1.09-3.02)	1.06 (0.73-1.55)
Muscle weakness	2.31 (1.31-4.08)	1.60 (0.93-2.75)
Cyclophosphamide	2.09 (1.01-4.33)	0.82 (0.36-1.91)
Mycophenolate mofetil	2.06 (0.04-1.09)	0.15 (0.02-0.93)^*^
Every 1-mg increase in prednisolone	1.08 (1.04-1.12)	1.06 (1.02-1.10)^*^

^*^Statistical significant. dcSSc, diffuse cutaneous systemic sclerosis; mRSS, modified Rodnan skin score; LV, left ventricular.

## Discussion

This cohort study followed up on the clinical data of patients with SSc in real-world practice. Our study is the first to address the factors associated with LV systolic dysfunction in patients with SSc by using longitudinal data routinely collected during follow-up. We included many SSc patients in our research because our institute has access to many SSc patients and a longitudinal dataset. As most internal organ involvement in SSc occurs in the first four years after the onset of the disease,^[16]^ we assumed that our lengthy follow-up period was sufficient to determine whether the patients experienced a decline in systolic function. Approximately 47.8% (384 of 803 cases in the database) were unsuitable for inclusion in the analysis. Three hundred and ten of the 384 cases were not regularly evaluated for cardiac involvement by echocardiogram. Patients with symptoms and signs of suspected LV systolic dysfunction (i. e., tachycardia or cardiac arrhythmia) were asked to undergo myocardial function evaluation or cardiac involvement assessment. Myocardial function is not routinely assessed in cases without clinical indications of cardiac involvement. The attending physician (s) would not have referred SSc patients who had never undergone an echocardiogram after the initial visit to a cardiologist if they did not exhibit clinical signs of LV systolic dysfunction. Patients excluded from the study might have resulted in an underestimation of the number of patients with LV systolic dysfunction if they had asymptomatic LV systolic dysfunction.

Approximately 10% of patients with SSc in our cohort had LV systolic dysfunction during follow-up, with a median disease duration of 8.5 years. Most were in the dcSSc subset, a severe form of SSc, and fibrosis predominates. Although, according to our analysis, inflammatory markers, specifically C-reactive protein, cannot predict LV systolic dysfunction, this finding suggests that the pathogenesis of LV systolic dysfunction may extend beyond inflammation, and that fibrosis may be a mechanism of LV systolic dysfunction. Consequently, it is unsurprising that LV systolic dysfunction was detected more frequently in dcSSc than in lcSSc patients. In addition, Tipparot *et al*. reported the myocardial inflammation according to the Lake Louise Criteria,^[18]^ from cardiac magnetic resonance imaging during the early onset of SSc (within 4 years of onset), specifically in patients with the dcSSc subset.^[8]^ The authors also found a combination of myocardial scar and myocardial inflammation from cardiac MRI in the same patients.^[8]^ Regarding the longer median duration of disease in our SSc patients compared to those in the study by Tipparot *et al*., our findings may represent the late complication of myocardial involvement in SSc, which should be associated with myocardial fibrosis rather than myocardial inflammation. Although we could not determine whether our patients had myocardial inflammation and/or fibrosis during follow-up or the duration of myocardial fibrosis because cardiac MRI was not a routine investigation in our cohort owing to budget constraints, the findings can provide insight into the natural course of myocardial involvement in SSc.

According to our analysis, the severity of skin thickness by the mRSS assessment strongly predicted LV systolic dysfunction and the clinical course of unimprovement LV systolic dysfunction. The mRSS assessment is a well-known surrogate marker of a poor prognosis and internal organ involvement.^[19]^ Previous research has linked higher mRSS and progression of mRSS to internal organ involvement, particularly pulmonary involvement.^[20,21]^ Furthermore, high mRSS has been linked to an elevation in cardiac enzymes in asymptomatic cardiac involvement^[9]^ and myocardial inflammation in the early onset of the disease.^[8]^ On the other hand, a study found a correlation between improvement of mRSS and less frequent new cardiac involvement.^[22]^ Our findings support the hypothesis that systemic—skin and internal organ—disease progression will occur in tandem. We hypothesized that if skin fibrosis were stopped or controlled, especially in the early stages of the disease, the involvement of internal organs would also be slowed or stopped. To date, no ideal medication can prevent the progression of skin thickening and/or involvement of internal organs at an early stage. Prior research on the early treatment of SSc with Tocilizumab, an interleukin-6 inhibitor, suggested that it would slow the progression of pulmonary involvement and stabilize pulmonary function; however, the primary endpoint of changing the mRSS relative to baseline was not met.^[23]^

We also found that muscle weakness was an additional predictor of LV systolic dysfunction and its progression of LV systolic dysfunction. This observation may be due to the same process of myopathy in SSc involving cardiac and skeletal muscle.^[24]^ Previous studies revealed an association between increased cardiac enzyme levels and muscle inflammation in SSc.^[25,26]^ Follansbee *et al*. evaluated the association between myocardial involvement and muscle disease in SSc and found that patients with myopathy were at risk of developing cardiac symptoms, primarily symptomatic cardiac arrhythmias, heart failure, and even cardiac death.^[24]^ The pathogenic link between cardiac muscle disease and myopathy or myositis in SSc has not yet been established. Nevertheless, once patients with SSc develop muscle weakness, myopathy, or an increase in muscle enzymes (creatine kinase), myocardial disease should be promptly evaluated and closely monitored.

However, the pathogenesis of LV systolic dysfunction remains unknown. Cardiac Raynaud’s phenomenon and/or microvascular disease of the heart is proposed as a leading cause of myocardial fibrosis and LV systolic dysfunction in SSc.^[27,28,29,30]^ Vasodilators such as calcium channel blockers should be a targeted treatment for the prevention of the progression of LV systolic dysfunction; however, neither anti-platelet nor calcium channel blockers can reverse the process by our analysis. This finding might indicate that the pathogenesis of LV systolic dysfunction is not solely due to microvasculopathy of the heart.

In our SSc patients, steroid treatment increased the risk of systolic dysfunction progression. According to Pussadhamma *et al*., steroid 30 mg/d reduces myocardial inflammation as measured by cardiac MRI in 8 of 12 SSc patients with early disease onset. Still, most of the cases in the study had normal left ventricular function.^[31]^ The authors also found that long disease duration was associated with nonresponse to steroid treatment among SSc with myocardial inflammation.^[31]^ Other studies showed the benefit of high-dose steroid treatment (methylprednisolone) for myocardial inflammation in an early-onset SSc, but all were case reports.^[32,33]^ In contrast, Hosenpud *et al*. found only marginal improvement in LV systolic function in 6 patients with myocardial inflammation when high-dose steroid therapy was combined with azathioprine.^[34]^

The efficacy of prednisolone for treating myocardial inflammation is controversial; however, prednisolone appears to be beneficial in the early onset of the disease, especially when inflammation is predominant. Nevertheless, steroid treatment is associated with several adverse and cardiovascular effects. 11β-Hydroxysteroid dehydrogenase type 1 or cortisone reductase is a crucial enzyme that activates the glucocorticoid receptor and mediates its effect according to the physiologic effect of steroids. The enzyme is also expressed in endothelial cells, where it causes vascular hypertrophy and fibrosis.^[35]^ In a cohort study, Wei *et al*. discovered that patients receiving high-dose exogenous steroids equivalent to prednisolone ≥ 7.5 mg/d had a 2.5-fold increased risk for cardiovascular events compared to those who received low-dose steroids.^[36]^ We hypothesized that the effect of steroids on cardiovascular disease could explain why steroids worsened LV systolic dysfunction in SSc. However, we did not investigate the duration of steroid exposure or perform coronary angiography in our patients with SSc to rule out coronary artery disease that could be related to steroid treatment. These findings led us to prescribe steroids with caution in SSc patients, particularly those with longer disease duration.

Mycophenolate may retard the progression of LV systolic dysfunction in SSc patients. According to our longitudinal data analysis, patients who received mycophenolate experienced less deterioration of LV systolic function than those who did not receive mycophenolate. Relatedly, mycophenolate has been reported to prevent graft rejection after heart transplantation. In a randomized, double-blind, controlled trial, Eisen *et al*. reported that mycophenolate could reduce graft loss and mortality in cardiac transplant recipients up to three years after transplantation.^[37]^ Although there is no clinical trial of mycophenolate for the treatment of myocardial disease in SSc, the experimental study described the effectiveness of mycophenolate against autoimmune myocarditis in both early and late treatment in a rat model. The area of inflammation by cardiac biopsy significantly decreased after receiving mycophenolate.^[38]^ In addition, mycophenolate was able to diminish myocardial ischemia in a rat model.^[39]^ The principle mechanism of action of mycophenolate is to inhibit inosine monophosphate dehydrogenase, an enzyme in de novo purine synthesis, and finally, the anti-proliferative effect; however, the effect and mechanism of mycophenolate on myocardium remain unclear. Further randomized controlled trials should be performed to support the efficacy of mycophenolate in preventing the progression of myocardial involvement in SSc.

Our study had some limitations. First, this was a single-center study; therefore, the generalized interpretation of the results should be made with caution. Second, cardiac enzymes, N-terminal pro-brain natriuretic peptide, and cardiac MRI were not routinely performed in the cohort. Third, specific autoantibodies of SSc were not tested for every patient; therefore, we could not investigate whether the autoantibodies predicted the clinical course of cardiac involvement in SSc. The strengths of our study include the following: a) a large number of SSc patients were included, resulting in a high power of analysis; b) the duration of follow-up was sufficient to assess the clinical course of LV systolic dysfunction in SSc; and c) the use of semiparametric Cox regression analysis with robustness clustering (by the patient’s cohort ID) for analyzing longitudinal data, resulting in a more appropriate interpretation and reliable conclusion. However, it is essential to externally validate the stability of the model.

## Conclusions

LV systolic dysfunction is uncommon in SSc, but most patients experience clinical unimprovement during follow-up. The severity of skin thickness indicates an increased risk of LV systolic dysfunction. High mRSS, steroid use, and high creatine kinase (CK) level were predictors of unimproved LV systolic dysfunction, whereas mycophenolate treatment might prevent the progression of LV systolic dysfunction. Vasodilators such as calcium channel blockers cannot change the clinical course of LV systolic dysfunction. Steroid prescriptions should be used with caution in patients with prolonged disease.

## Supplementary Material

Supplementary Material
