# Supplementary material for: Clinical courses and predictors of left ventricular systolic dysfunction in systemic sclerosis: A cohort study
Source: Rheumatol Immunol Res. 2024 Jul 15;5(2):107–16. doi: 10.1515/rir-2024-0014 (PMC11248551; doi:10.1515/rir-2024-0014)
Supplement: Supplementary file 1 — Supplementary Material [file rir-2024-0014_sm.pdf]

Supplementary Materials

Supplement table 1. Clinical characteristics of patients with LV systolic dysfunction on the last visit.

**Data**	***N* = 35**
Female (%)	23 (65.7)
Age of onset (years) mean ± SD	51.5 ± 10.1
Age on the date of LV systolic dysfunction detection (years) mean ± SD	57.9 ± 9.2
Age at last follow-up (years) mean ± SD	59.7 ± 8.9
Duration of disease on the date of LV systolic dysfunction detection (years) mean ± SD	6.2 ± 4.5
Duration of follow-up (years) median (IQR)	8.21 ± 5.2
dcSSc subset (%)	56 (74.3)
WHO functional class (%)	
I	8 of 28 (28.6)
II	15 of 28 (53.6)
III	5 of 28 (17.9)
IV	0
mRSS (points) median (IQR)	4 (0-18)
Raynaud's phenomenon (%)	21 (60.0)
Digital ulcer (%)	7 (20.0)

Digital gangrene (%)	2 (5.7)
Telangiectasia (%)	18 (51.4)
Calcinosis cutis (%)	3 (8.6)
Salt and pepper skin (%)	21 (60.0)
Edematous skin (%)	3 (8.6)
Tendon friction rub (%)	11 (34.4)
Hand deformity (%)	15 (42.9)
Arthritis (%)	2 (5.7)
Muscle weakness (%)	2 (5.7)
Esophageal involvement (%)	19 (54.3)
Stomach involvement (%)	7 (20.0)
Intestinal involvement (%)	7 (20.0)
Pulmonary fibrosis (%)	15 (42.9)
Pulmonary arterial hypertension (%)	12 (34.3)
Hemoglobin (g/dL) mean ± SD	11.7 ± 2.0
Creatinine (mg/dL) median (IQR)	0.9 (0.8-1.1)
Albumin (g/dL) mean ± SD	3.8 ± 0.6
CRP (mg/L) median (IQR)	1.2 (0.8-53)
Creatinine kinase (U/L) median (IQR)	110.5 (29-206)
Treatment	
Aspirin (%)	23 (65.7)
Nifedipine (%)	19 (54.3)

Domperidone (%)	18 (51.4)
Cyclophosphamide (%)	5 (14.3)
Mycophenolate mofetil (%)	5 (14.3)
Hydroxychloroquine (%)	2 (5.7)
Prednisolone (%)	24 (68.6)
Low dose	10
Moderate dose	14
High dose	0

SD, standard deviation; IQR, Interquartile range; dcSSc, diffuse cutaneous systemic sclerosis; WHO, World Health Organization; ESR, erythrocyte sedimentation rate; CRP, C-reactive protein.
